# A richly annotated dataset of co-speech hand gestures across diverse speaker contexts

**DOI:** 10.1038/s41597-025-06020-6

**Published:** 2025-11-05

**Authors:** Laura B. Hensel, Stephanie Cheng, Stacy Marsella

**Affiliations:** 1 University of Glasgow, School of Psychology and Neuroscience, Glasgow, G12 8QB United Kingdom; 2 Northeastern University, Khoury College of Computer Sciences, Boston, MA 02115 USA

**Keywords:** Scientific data, Human behaviour, Databases, Communication

## Abstract

Hand gestures form an integral part of human communication and their complexity makes their study and generation difficult. Here, we present a dataset comprising 2373 annotated gestures, designed to facilitate in-depth analysis of human communication. We captured these gestures from nine speakers across three distinct categories: University lecturers, Politicians, and Psychotherapists. The annotations encompass various aspects, including gesture types (e.g., metaphoric, iconic), descriptive terms characterizing each gesture (e.g., ‘sweep’, ‘container’), and their corresponding verbal utterances. The dataset also includes detailed physical properties such as hand height, distance to the body, arm angle, hand configuration, palm orientation, repetitions, size, and speed, alongside 3D pose tracking data. Where possible, video recordings provide additional multimodal context. Notably, we identified several previously undocumented lexemes, expanding the current lexicon of gesture research. This dataset offers a valuable resource for studying human communication, training models for gesture recognition and generation, and designing socially intelligent virtual agents.

## Background & Summary

Hand gestures are a fundamental part of human communication^1^. They enhance the listener’s understanding^2^, recall^3,4^, and interpersonal liking^5^ all while aiding the speaker’s speech production^6–10^ (but see^11^). Consequently, gestures are of interest to many scientific fields: Psychologists seek to understand their communicative role^1,5,12,13^ and cognitive correlates^14–17^. Computer scientists seek to artificially replicate and formalize the behavior^18–20^, often to enhance virtual human’s communicative skills. Linguists seek to understand gestures’ role in language acquisition^21–23^, language evolution^24,25^, and multi-modal interaction^26–29^.

However, the systematic analysis of human gestures remains challenging, in part because gestures involve multiple articulators creating a vast, high-dimensional information space. Each arm and hand can move at 18 different joints, each with as many as five degrees of freedom, including flexion, extension, abduction, adduction, and rotation. Additionally, both arms can, and frequently do, move independently to produce gestures. Additionally, unlike facial expressions, which can be decomposed into standardized Action Units^30^, gestures lack a widely adopted formal decomposition system, making them more difficult to describe and interpret consistently.

To constrain this information space, the focus is often placed on specific categories of gestures. There are six broad gesture categories commonly identified in the literature^31^: Iconic gestures wherein the gesture imitates the physical form of an object, for example the roundness of a ball. Metaphoric gestures which are physical representations of abstract ideas, such as a container gesture that represents groups of people. Deictic or pointing gestures which also include abstract deictics, i.e., pointing gestures to something that is not physically present. Beat gestures are typically rhythmic and repeated movements that do not convey clear semantic content; instead, they serve to mark discourse structure or emphasize prosodic features. And finally, emblematic gestures which carry specific cultural meaning and can convey information independently of speech, such as the thumbs up gesture. In addition, humans also produce adaptor and manipulation gestures – touching the own body and manipulating other bodies and objects, respectively^31^.

However, while all people use such gestures, they differ drastically in *how* they use them. People differ in their gesturing frequency and the types of gestures they use^15,32^. Moreover, humans also gesture for many different reasons and will adjust their gesturing based on their audience and the context^33^. For example, people use fewer and smaller gestures when talking to a social superior as compared to a friend^34^ and change gestures based on listener knowledge^35,36^, shared representational space^37^, and the type of information conveyed (positive *vs*. negative)^38^. In high-stakes situations, people increase their gesturing rate^14^. This further translates into gesturing differences between social and professional context. For example, language teachers produce more, larger, and more illustrative (iconic/deictic) gestures^39^ and increase gesturing frequency when teaching difficult concepts^40^ which in turn helps students to learn^3^. Similarly, politicians strategically use gestures^41–43^, exploiting the fact that they make speech more persuasive^44^.

To address these complexities, datasets often focus on one or two of the gesture categories, such as iconic or emblematic gestures, often using just a few example gestures as demonstrated by the illustrative (but non-exhaustive) selection of datasets in Table 1. Emerging evidence also suggests that some of the more comprehensive datasets have data quality issues^45^. Additionally, because these datasets are created either for machine learning or for psychology but seldom both, they usually contain only one type of information - video or annotation data. However, the symbiotic relationship between psychology and human-centered computing is growing^46,47^ and calls for datasets that can speak to questions of both fields. While video data is sufficient for machine learning problems, it often falls short when trying to explore psychological and linguistic phenomena associated with gestures. These may be embedded in the interplay between hand shape, palm orientation, gesture type, associated speech and the larger context of the interaction.

**Table 1:** Examples of hand gesture datasets.

Dataset	Medium	Description
HANDS^69^	video frames	29 static gestures primarily emblems produced by 5 people
3DIG^70^	video and motion capture	1739 dynamic iconic gestures referring to 20 objects produced by 30 people
BEAT^71^	motion capture	number of gestures not reported 76-hour 3D motion capture produced by 30 people semantic relevancy and emotion categories annotations
NEMO^72^	video and motion capture	3715 dynamic gestures 2D & 3D motion capture produced by 428 people iconic gestures referring to 35 objects
M3D-TED^73^	annotations	1139 gesture strokes produced by 5 English speakers 23 minutes of annotated referential and non-referential gestures
EcoLang^74^	video and annotations	produced by 78 dyads naturalistic gestures during adult-child and adult-adult interaction
Talking With Hands Dataset^75^	motion capture	no number of gestures reported 50-hour 3D motion capture produced by 50 people collaborative gestures captured during conversation
EGGNOG^76^	video and motion capture	20 dynamic gestures produced by 40 people 8 hours of video gestures during collaborative object-oriented task
Speaker-Specific Gesture Dataset^77^	videos and motion capture	dynamic gestures 144-hour 2D motion capture produced by 10 people (lecturers, TV show hosts, televangelists)
SaGA^78^	videos	1764 gestures detailed gesture annotations produced from 25 dialogs in direction-giving task iconic and deictic gestures
Dutch dyadic dataset^79^	video and annotations	439 gestures produced by 34 dyads in Dutch naturalistic gestures in conversation

For each dataset, we list its medium (video, motion capture) and give a brief description of its main properties including the type and number of gestures included.

Here, we introduce a new dataset, GESRes (Gesure Exploration and Synthesis Resource), consisting of 2373 gestures of three different groups of speakers that represent a variety of different communication roles and intents: University lecturers, politicians, and psychotherapists. Lecturers aim to increase understanding and recall, politicians want to persuade their audience, and psychotherapists seek to create an inviting space and increase their clients’ understanding of themselves. For each individual speaker – 9 in total – we annotated between 17 and 48 minutes of video footage. We included every observable (i.e., not obscured) gesture, including all seven categories: iconic, metaphoric, deictic and abstract deictic, beat, emblem, adaptor. Finally, based on our annotations, we collated a gesture lexicon which includes 149 descriptions of gestural form (lexemes) thereby significantly extending the variety of lexemes described in the literature.

## Methods

To collate this comprehensive set of gestures, we sought to include a variety of speakers from different professions and then annotated video footage of each speaker. Gesture annotation approaches vary in how much physical and semantic information they code for. Some approaches are entirely form-based and highly detailed, often including spatio-temporal properties of the gestures with many degrees of freedom^48,49^. However, with increasing detail, the interpretability of the resulting data decreases^50^ as complex spatio-temporal information is hard to parse. Subsequent work therefore often simplified the annotation of these spatio-temporal properties^50,51^. Other work takes a more functional approach while still including some temporal information^50,52^. That is, annotations in these approaches do not only refer to physical properties of gestures but encode semantic information as well. Finally, some approaches comprise entirely free-form descriptions of hand gestures^53^. While such approaches are ethnographically rich, they make systematic analysis of gestures and their use for gesture generation difficult. Here, we aimed to find a middle ground between completely form-based annotation schemes that can make data interpretation difficult and more interpretive, functional approaches which can hinder physical comparisons of gestures.

### Video data

We sourced video data from a variety of public and licensed materials (see *Gesture video segments* below and the document ‘Licensing_information.pdf’ included with the dataset^54^). In total, we included nine individual English speakers from the United States: Three University lecturers (1 female, 2 male), three politicians (2 female, 1 male of which 2 were Democrats and 1 Republican), and three psychotherapists (2 female, 1 male). For brevity, from here on and in the dataset itself, we refer to psychotherapists as clinicians and University lecturers as lecturers. The lecturers delivered public lectures, the politicians gave televised speeches, and the clinicians conducted mock-therapy sessions with actors. While all speakers produced gestures in naturalistic settings, we acknowledge that the degree of spontaneity may vary across contexts – for instance, politicians may have received rhetorical or media training, whereas the clinicians were conducting unscripted therapy sessions (though the patients were actors) and thus produced spontaneous gestures, similar to the unplanned gestures observed in the lecturers’ public talks. We selected speakers based on the following criteria:

- Video length available with a minimum set at 15 minutes
- Video quality and visibility of hands and arms during the majority of the video. Note that we did not exclude videos for changing camera angles
- Roughly equal distribution of male and female speakers
- Representation of political parties for politicians and subjects for University lecturers
- Native English speaker

We thus annotated a total of 12 videos, covering 3 hours and 46 minutes of footage, measured up to the final gesture annotated in each clip. Table 2 gives an overview over the speaker information, including length of annotated video per speaker. We stopped annotation once reaching either a minimum of 15 minutes annotated or once we had reached at least 100 suitably varied gestures. Our goal was to ensure that each speaker’s dataset included a diverse range of lexemes. Rather than aiming for uniform clip lengths or total gesture counts across speakers, we prioritized depth over breadth – annotating longer stretches of video per individual speaker to capture the richness and variability within their gestural repertoire. In cases where we reached the 15-minute or 100-gesture mark but observed limited diversity of lexemes (typically around five or fewer distinct lexemes), we continued annotating until we felt the sample reflected a broader range of gesture types. In most cases, we required between 20–30 minutes of video to reach a large enough number of different gestures.

**Table 2:** Speaker information.

Speaker ID	Details	Number of videos	Total length (minutes:seconds)
Lecturer1	Male; Subject psychology	1	48:30
Lecturer2	Male; Subject politics	1	29:56
Lecturer3	Female; Subject law	1	24:53
Politician1	Female; Democrat	2	17:20
Politician2	Male; Democrat	2	20:00
Politician3	Female; Republican	2	19:37
Clinician1	Female	1	16:39
Clinician2	Male	1	24:08
Clinician3	Female	1	25:06

Summary of annotated videos. For each individual speaker (column 1) we show their sex and, where appropriate, political party or taught subject (column 2), the number of videos annotated (column 3), and the total time of the annotated footage in minutes and seconds (column 4).

### Video annotation

To annotate these videos, we used the annotation tool ELAN version 6.7^55^. We adapted the annotation system by Kipp *et al*.^50^, but did not include separate annotations for individual gesture phases (e.g., preparation, stroke, retraction). Instead, we annotated each gesture as a whole gesture phrase^53^, encompassing all observable phases. In many cases, these gesture phrases occurred as part of continuous gesture sequences, where the hands did not return to rest between gestures. This structure closely aligns with Kendon’s description of the G-Unit^53^.

Specifically, we created 16 annotation tiers, each corresponding to a specific gestural attribute as listed below, to describe the form and characteristics of each annotated gesture phrase. We also included an additional ‘notes’ tier for any other remarks. We created annotation dictionaries, i.e., drop-down selection menus, for each physical attribute (handedness, height, distance, radial orientation, arm swivel, trajectory, palm orientation, hand shape, movement size, movement speed) and for gesture type. This ensured consistency in naming conventions across annotations. The dataset is accompanied by an annotation manual^54^ that details each annotation step and considerations for individual tiers. This serves both as a record of our annotation approach and as a resource for anyone intending to produce additional annotations. The following are the attributes the dataset includes:

- **Gesture Type**: Iconic, metaphoric, emblematic, deictic, abstract deictic, beat, adaptor
- **Lexical affiliate**: The utterance the gesture was associated with (i.e., the part of the utterance the gesture was semantically related to; for beat gestures, based on co-occurrence only)
- **Lexeme**: The general gesture label such as sweep (a broad sweeping movement of the hand/arm, often moving laterally) or container (a gesture that mimics holding or enclosing an object or space)
- **Handedness**: Which hand produced the gesture
- **Height**: The height, relative to the body, at which the gesture was produced (see Fig. 3)
- **Distance**: The distance between the hand(s) producing the gesture and the body (see Fig. 3)
- **Radial orientation**: The orientation of the arm radially to the body (see Fig. 3)
- **Arm swivel**: The angle between the arm and body (see Fig. 3)
- **Trajectory**: The path of the gesture (straight, curved, circular)
- **Palm orientation**: The direction the palm of the hand was facing
- **Hand shape**: The hand shape (see Fig. 4 for illustrations of individual hand shapes)
- **Movement size**: The overall size of the gesture relative to other instances of this specific gesture
- **Movement speed**: The overall speed of the gesture relative to other instances of this specific gesture
- **Repetition**: The number of times the speaker repeated the gesture
- **Unit**: We use the term unit to refer to sequences in which the hand(s) do not return to a resting position between gestures but instead transition directly into another gesture. This conceptualization closely aligns with Kendon’s G-Unit^53^. In some cases – though not always – these continuous gesture sequences convey related or evolving meaning and may reflect a higher-level ideational structure^56^.
- **Polysign**: This denotes instances where the speaker carried out two clearly defined lexemes in conjunction.

As lexical affiliates alone can be very short, we subsequently added context to each lexical affiliate by including a short segment before and after each lexical affiliate in a separate annotation column. To obtain this, we first used automatic speech-to-text transcription with the Whisper model (version “base”) in Python. This involved extracting audio from the videos and applying Whisper’s default English language and alignment models via its transcribe() method, using default batch size parameters. Next, a researcher carefully checked the resulting transcripts for errors and made corrections where necessary. Finally, for each annotation, we extracted the transcript segment within which the specific lexical affiliate fell and stored it in the column ‘combined_content’.

#### Gesture types

Additionally, although gesture types are not mutually exclusive^57^, the annotator selected the single type that best captured each gesture’s primary function, following the criteria outlined in the annotation manual. When a gesture could plausibly fit multiple categories, the annotator prioritized the type with the strongest contextual fit. Beat gestures were assigned only when no other type was appropriate, with the absence of related semantic content as the primary criterion. Other features – such as repetitive up-and-down movement – were not used as defining criteria, as they may also occur in non-beat gestures. To ensure consistency, the annotator re-evaluated all gesture-type labels after completing the initial annotations, and consulted a second expert in cases of uncertainty to reach a consensus.

#### Lexemes

As noted above, characterizing gestures solely by their overall physical characteristics such as movement pattern in 3D space across time quickly leads to uninterpretable results^50^. To account for this, we categorized gestures by their overall physical form also referred to as ‘lexemes’. For example, a hand sweeping through the air is called *sweep*. Lexemes may contain semantic information about the gesture. However, as we primarily aimed to describe gestures physically, we focused on the lexemes’ generalized physical properties. Where possible, we used existing lexemes from the literature, even if their names include pragmatic or semantic information, because they offer a shared vocabulary for recurring physical gesture patterns. For this, we first collated lexemes from the literature^50,56^ for a set of 69 lexemes. We excluded lexemes that were too specific or were covered by another lexeme. For example, *chide* and *attention* share two main identifying features: Holding up the hand and shaking it. They differ, however, in their semantic meaning resulting in separate classification from Kipp *et al*.^50^. As we sought to focus on the shape of the gestures at this stage, we only included one of the two lexemes. Note that we did not observe instances of all of these 69 lexemes in our dataset. However, during annotation, we created an additional 97 lexemes as follows: If a gesture did not conform to the descriptions of any existing lexemes, we created a new data entry with a full description of the gesture including an example video timestamp. In some cases, these additions are simply an added description of the movement of an already existing lexeme, for example “container (moving left)”. In many other cases, however, the added lexemes represent new movement patterns.

To ensure that we consistently categorized gestures as specific lexemes, upon completing all annotations, we extracted individual video clips associated with each lexeme and compared them. Specifically, two researchers jointly viewed all video segments of a given lexeme and re-evaluated the visual similarity between these video segments. Where necessary, we changed annotations at this stage. In total, the final dataset therefore included 149 different lexemes, comprised of 54 lexemes previously described in the literature and 97 lexemes not, to our knowledge, previously described. Note that many of these 97 lexemes represent variations or extensions of established lexemes, such as the expanding container. We describe the complete set of 149 lexemes in a dedicated file located in the dataset repository^54^.

#### Handedness

A large proportion of gestures in the dataset are two-handed and symmetric. Specifically, if the handedness tier takes the value ‘both’, the gesture is assumed to be two-handed and symmetric, unless another tier – most commonly palm orientation – indicates asymmetry by explicitly providing different values for each hand, or unless the notes tier includes a comment indicating asymmetry. In such cases of symmetric two-handed gestures, the single value annotated for a tier (e.g., palm orientation or hand shape) applies to both hands. For example, in a *container* gesture, both hands are typically palm-shaped and facing in – this is captured as a single palm orientation value (*in*), which applies to both hands.

However, the annotation scheme also supports asymmetric two-handed gestures. For example, annotators could assign palm orientation values like *left down, right up* when the hands had clearly distinct orientations. In cases where a particular asymmetric form occurred repeatedly across the dataset, it is defined as a unique lexeme, with detailed descriptions included in the ‘Lexemes.csv’ file (e.g., the *space* gesture, in which one hand points toward the other). These entries specify which hand moves, the relative positioning, and orientation, allowing accurate interpretation of asymmetry in recurring forms.

For the very small number of non-recurring asymmetric gestures that could not be fully described using the standard scheme, annotators included free-text notes in the “Notes” column of the annotations to document what occurred.

We recommend users consult both the annotation manual and the ‘Lexemes.csv’ file to understand how specific hand configurations and movement patterns are annotated^54^, particularly in two-handed or asymmetric gestures. Together, these resources provide the necessary context for accurate and reproducible analyses of gesture form.

### Gesture video segments

Upon completing the annotations and lexeme consistency checks, we re-rendered the video segment for each individual annotation by extracting the corresponding portion from the longer video based on the annotation’s start and end times. Before rendering, we cropped the frames to remove non-target individuals and applied a standardized naming convention: annotationNumber_speakerID_lexemeOrAdaptor. Note that some of the videos included are used under explicit licenses obtained from rights holders, while others are publicly available but not formally licensed for redistribution. Where licensing restrictions apply, we provide links to publicly available versions of the videos, along with code to extract relevant segments. Licensed videos are included in the dataset only where permitted, with proper attribution where required. Full details for each individual video are included in the document ‘Licensing_information.pdf’.

### Gesture landmark estimation

**Fig. 1:**
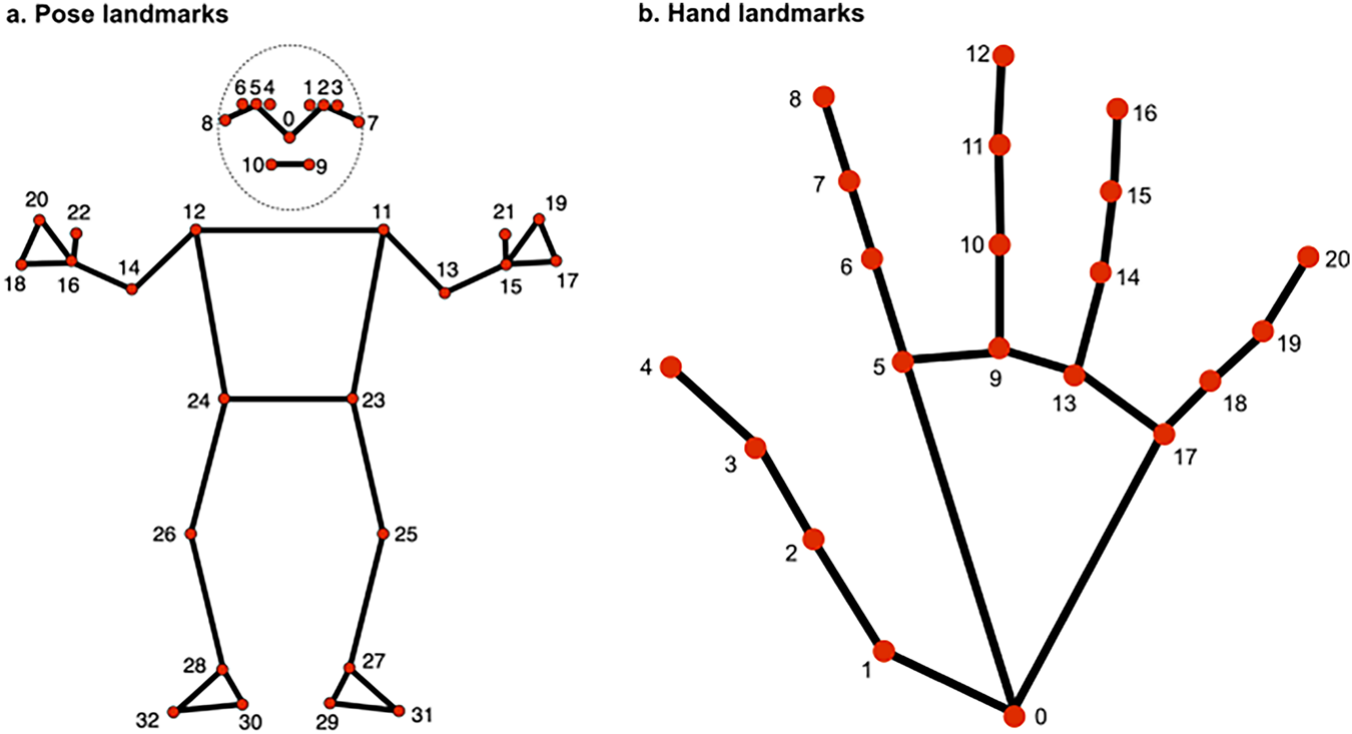
Pose (**a**) and hand (**b**) landmarks. Stick figure representations show the position of each of the 54 individual landmarks estimated by MediaPipe as red points.

Finally, to supplement the gesture form information provided by our annotations, we additionally extracted 3D positions of 33 pose landmarks and 21 hand landmarks for both hands of each annotation video using Google’s MediaPipe^58^. Specifically, we used the pose landmarker (heavy) and HandLandmarker (full) pre-trained models to estimate the 3D position of each landmark for each of the 2373 individual gesture videos. However, due to technical limitations and challenges in hand detection (e.g., occlusions, poor lighting, or absence of hands in certain frames), not all annotations in the dataset include hand-tracking results. The full code is available on the Open Science Framework^54^. Figure 1 shows the placement of individual landmarks as red points (portions of this figure are modifications based on work created and shared by Google and used according to terms described in the Creative Commons 4.0 Attribution License).

## Data Records

The GESRes dataset is available from the Open Science Framework (OSF)^54^ and can be accessed at 10.17605/OSF.IO/9ZKFM. The annotation dataset is shared openly under a CC BY license, while the associated videos are shared under a CC BY-NC-SA license, in accordance with the terms set by the original video owners. For videos without explicit reuse licenses, we provide external links instead of hosting the files directly. Table 3 gives an overview of the specific content of the repository, including the names of files and folders. We provide the dataset in two separate formats: as two comma-separated value (CSV) files containing annotations and pose tracking results, respectively, along with an associated codebook in the form of a Microsoft Excel Spreadsheet, as well as a JavaScript Object Notation (JSON) file. The JSON version includes the 2373 annotations, pose tracking data, codebook, and additional metadata, such as the creation date and detailed information about individual videos, including the posture of speakers (sitting *vs*. standing), whether they were holding objects such as microphones, and the overall video context (e.g., speech at congress; year).

**Table 3:** Dataset content.

File or folder name	Description
GESRes_dataset.csv	Annotation data, including the 16 gestural attributes described above as well as the following information: A unique identifier number Annotation start and end times in milliseconds and hh:mm:ss format Clip duration in seconds The video ID corresponding to the full video clip The video ID corresponding to the annotation clip The speaker group (Lecturer, Politician, Clinician) The speaker ID (e.g., Politician1, Politician2 etc.) The longer utterance spoken just before, during, and after the annotation utterance
GESRes_codebook.xlsx	A code book detailing the information contained in each column of the annotation data.
GESRes_dataset.json	A JSON file containing the full dataset, metadata including video information, and the full codebook.
lexeme_descriptions.csv	A file containing the names and detailed descriptions of each lexeme.
gesture_annotation_template.etf	An ELAN template file. This can be used to import tier structure into ELAN for annotation.
GESRes_annotation_manual.pdf	An annotation manual detailing exact annotation procedures.
Licensing_information.pdf	Contains detailed information on the license types for each video and the availability of non-licensed videos.
01Gesture_videos	Contains individual video clips for each hand gesture (licensed videos only) including audio.
02Full_videos	Contains full videos where licensing allowed. This allows for other researchers to add their own annotations or try out our annotation approach.
03Transcripts	Contains Transcripts for each video.
04Tracking_data	Hand tracking data in 3D for 33 pose and 21 hand landmarks.
05Code	Folder containing all code used to produce and evaluate the dataset. See ReadMe.rtf file for description of scripts.
ReadMe.rtf	A file listing all files and folders in the directory, including listing individual analysis scripts.

Details of the content of the dataset, stored on the OSF^54^. For each file or folder, we specify the content, including its use-cases where appropriate.

The JSON format is particularly advantageous for integrating the dataset into modern workflows that require structured data. It allows seamless compatibility with various programming languages and tools commonly used in data science, such as Python, R, and JavaScript. Furthermore, JSON’s hierarchical structure enables efficient storage and querying of nested metadata, such as speaker-specific details and contextual information about individual videos. This makes it ideal for applications requiring metadata-driven analysis or visualization, and for researchers who wish to customize their data parsing or processing pipelines. The full dataset, including all metadata, code, and videos has a size of 6.7 GB.

### General data overview

**Fig. 2:**
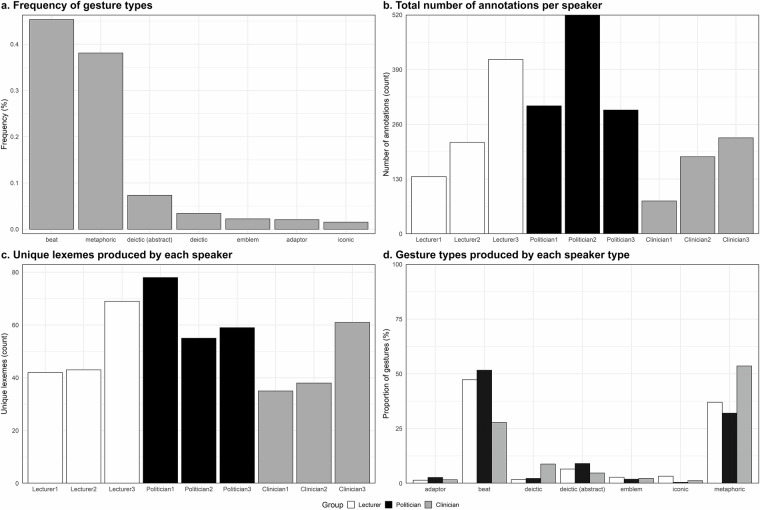
General data overview. Bar plots show several properties of the dataset including (**a**). the proportion of different gesture types across the whole dataset, (**b**). the total number of annotations per speaker, (**c**). how many unique lexemes each speaker produced, and (**d**). the percentage of the different gesture types (e.g., beat, iconic) each type of speaker (Lecturer, Politician, Clinician) produced.

Figure 2 gives a general overview of the gestures within this dataset. Of the 2373 gestures, the majority (45.74%) are beat gestures, followed by metaphoric gestures (37.56%) as seen in Fig. 2a. However, in many cases beat gestures also consisted of lexemes other than *beat*. Furthermore, although we took care to annotate similar amounts of data for each speaker, the final number of annotations for each speaker varied as shown in Fig. 2b. This is also due to the generally higher gesture frequency of some speaker types (e.g., politicians) compared to others. In addition, the data shows clear differences in (a) the variety of each speaker’s gestural repertoire, i.e., the number of unique lexemes each speaker produced (Fig. 2c), and (b) the types of gestures each group of speakers (Lecturer, Politician, Clinician) produced most frequently (Fig. 2d).

### Physical properties

**Fig. 3:**
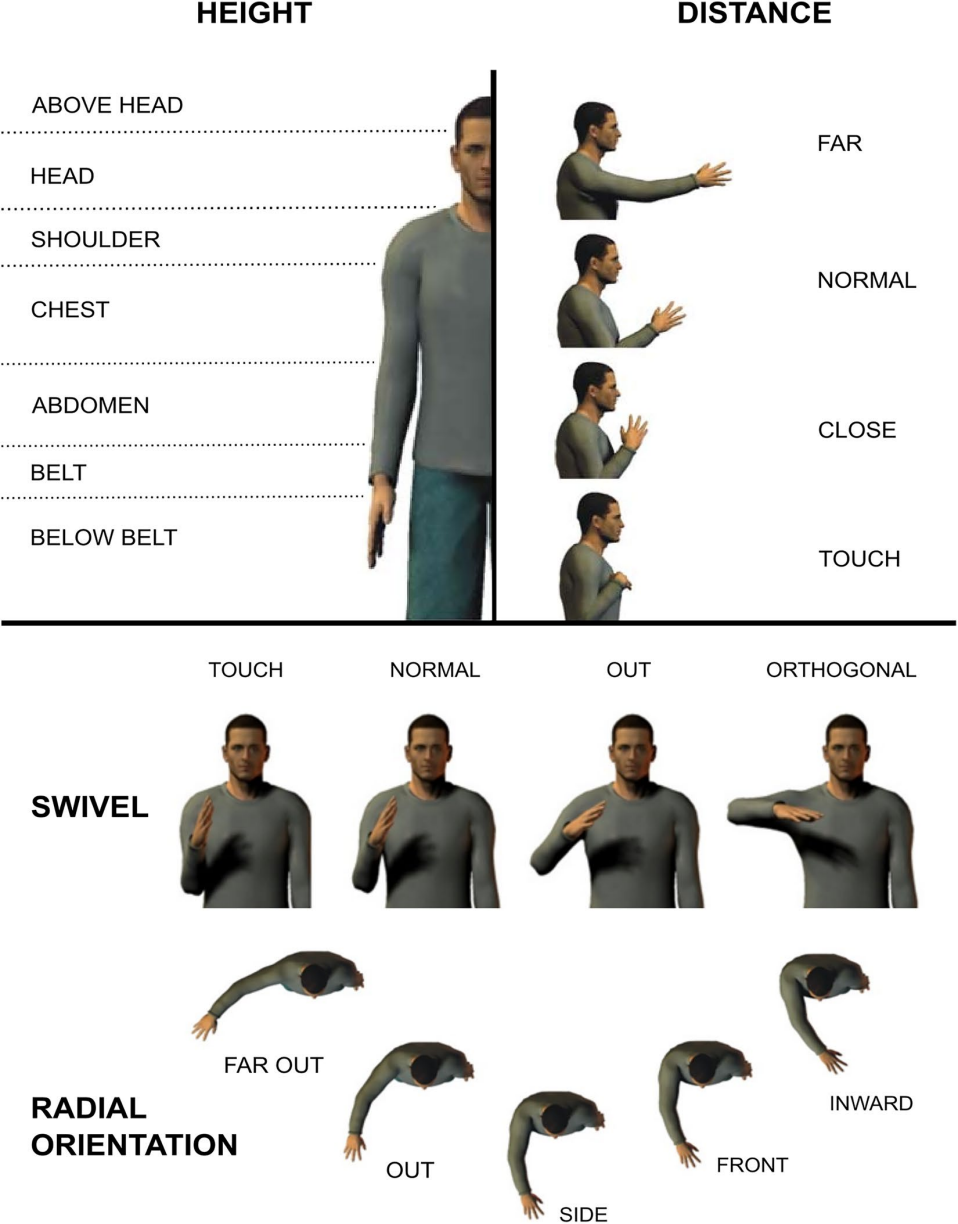
Physical properties. Illustration of four physical properties: Height, distance, swivel, and radial orientation. For each, we illustrate the values the physical property can take. Adapted with permission from^50^.

**Fig. 4:**
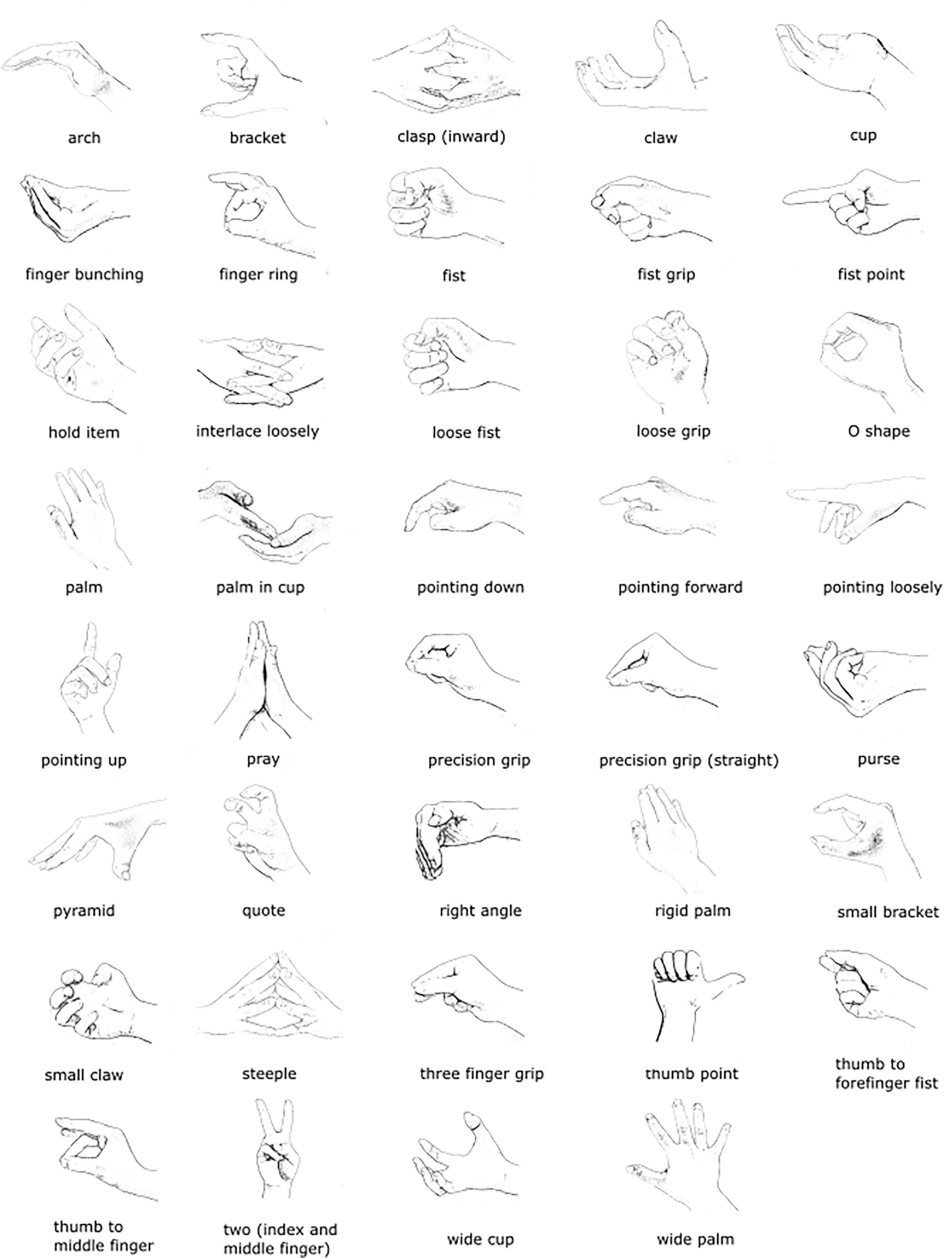
Individual hand configurations. Each image represents one of 39 hand configuration with its corresponding label.

Beyond gesture types and lexemes, the dataset contains annotations of 11 physical properties. While some of these, such as the number of repetitions or the handedness (left handed, right handed, two-handed) are readily interpretable, some will benefit from a more detailed explanation. In particular, the hand position properties – height, distance, radial orientation and arm swivel – can appear complex at first glance. Figure 3 illustrates the possible values these physical properties can take. For example, arm swivel refers to the angle between the upper body and the arm and can take values ranging from touch – the arm touches the body – to orthogonal – the arm is at an orthogonal angle to the upper body. Additionally, we recorded 39 individual hand configurations which we illustrate in Fig. 4. Note that we also observed an additional three types of gestures where hand configurations changed (e.g., *fist to palm*). We annotated these accordingly. The codebook includes all 42 hand configuration labels^54^. Most of these such as *finger ring*, *fist*, or *precision grip* have previously been described in the literature^50,53,56,59,60^. However, in some cases, we observed hand shapes that were similar to those previously described but with small yet potentially important physical differences. For example, in addition to *fist*, we added *loose fist* which refers to a more relaxed fist.

## Technical Validation

To evaluate the reliability of the dataset, we tested the annotation quality in three ways:

### Inter-rater reliability

**Fig. 5:**
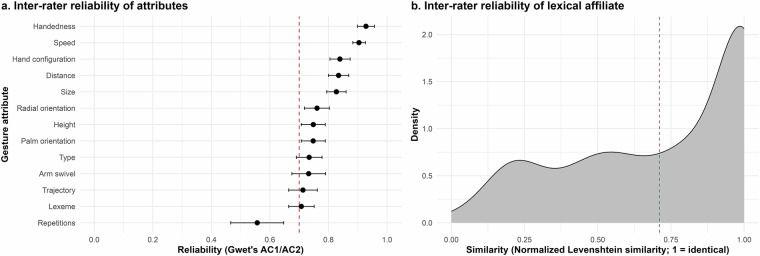
Inter-rater reliability of annotations. We compared the annotations of one additional annotator to the original annotations using Gwet’s AC1/AC2 (**a**) and normalized Levenshtein similarity for lexical affiliates (**b**).

First, to evaluate the consistency of the gesture annotation scheme, we conducted an inter-rater reliability analysis involving a previously trained annotator. This annotator, who is one of the co-authors, has been working with gestures for two years and has prior experience conducting multimodal annotations, including both speech and gesture data, making them well qualified for this task. They had originally contributed to the dataset by partially annotating one video (Politician 3, video 2), but for this analysis, they re-annotated the other video of the same speaker (Politician 3, video 1). Specifically, they re-annotated the first 50 gestures for each of the nine gesturers, resulting in a total of 450 re-annotated gestures. Because their original annotations, which had been reviewed by the main annotator, only covered a subset of the attributes (excluding, for example, gesture type) and given the time elapsed since the original annotation effort and the inherent complexity of the task, the annotator received an extensive re-training session. Together with the first author, they reviewed the annotation manual and jointly annotated a practice video not included in the analysis, discussing each annotation tier and the range of coding options in detail. The annotator then independently annotated all relevant attributes for the selected gestures. We then compared these annotations to the original annotations using Gwet’s AC1 for categorical variables and Gwet’s AC2 for ordinal variables (Fig. 5a). Gwet’s AC1/AC2 can be interpreted as the conditional probability that two raters will agree, assuming no agreement occurs by chance, offering a more stable alternative to traditional chance-corrected measures like Cohen’s Kappa^61^. For the lexical affiliate tier, we calculated the normalized Levenshtein similarity (1 - distance) between the original and re-annotated lexical affiliate entries (Fig. 5b). Higher values here, too, indicate higher similarity. Results showed high inter-rater agreement for all variables with AC1/AC2 coefficient values ranging from 0.71 to 0.93 (mean = 0.80) and generally high similarity for lexical affiliates.

### Intra-rater reliability

When interpreting the preceding results, it is crucial to consider that the utility of this dataset hinges on its consistency – specifically, the degree to which annotations are applied consistently by an annotator across the dataset. Consistency ensures that patterns in the data are meaningful and not obscured by variability in how individual annotators interpret or apply the annotation scheme. To assess this, we evaluated the intra-rater reliability, which measures the stability and reproducibility of an annotator’s ratings across multiple instances. High intra-rater reliability strengthens the dataset’s overall validity, supporting its use for downstream analyses and applications.

**Fig. 6:**
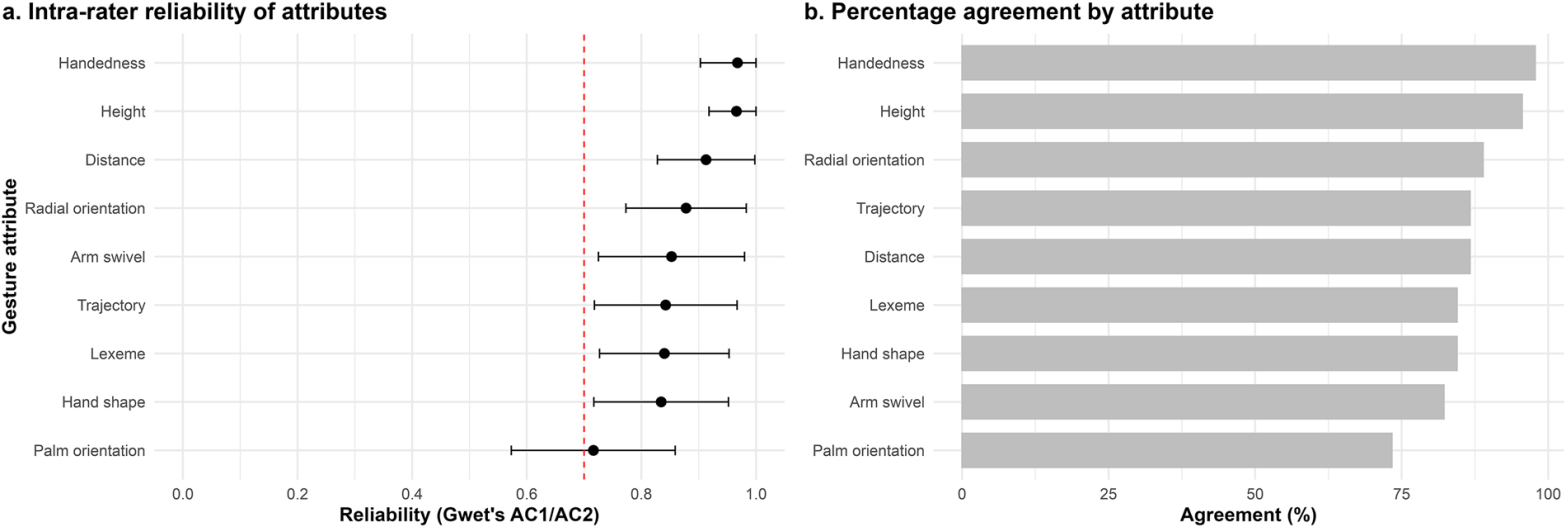
Intra-rater reliability of annotations. (**a**). Points and error bars show reliability measured via Gwet’s AC1/AC2 and (**b**). A bar plot shows percentage agreement. Gesture attributes are ordered via strength of agreement.

Here, a single expert annotated the entirety of the dataset, with a few exceptions where a second expert supplied annotations that the primary expert thoroughly verified. To directly measure intra-rater reliability, the original expert re-annotated 45 gesture clips approximately six months after completing the initial annotations. These clips comprised a subset of the annotations including five randomly selected annotations from each of the nine speakers while ensuring a wide distribution of lexemes. Based on these new annotations, we measured intra-rater reliability via Gwet’s AC1/AC2. As Fig. 6a shows, the annotator achieved high intra-rater reliabilities of between 0.72 and 0.97 with a mean of 0.87. These results suggest consistently high annotation quality across tiers.

These results are supported by percentage agreement values, as shown in Fig. 6b. Notably, the lowest agreement was observed for the palm orientation tier, which also had the largest number of possible values. For example, similar values such as *in* and *up-in* were not treated as equivalent, making this a particularly stringent test of reliability. Overall, these findings underscore the robustness of the dataset’s annotations and confirm their consistency across the dataset.

### Lexeme groups

Finally, we evaluate whether the clusters created via our lexeme categorizations correspond to actual physical similarities between gestures. To do so, we first calculated pair-wise similarities of annotations based on the 3D pose tracking data via Dynamic Time Warping (DTW)^62^. Dynamic Time Warping is designed to estimate the (dis)similarity between temporal sequences which may vary in their speed and length. We specifically focused on the 3D tracking data of the wrists as they serve as a good proxy for the movement of the arm and hand. This initially yielded a 2373  × 2373 matrix of (dis)similarity values. To improve data quality, we reduced the size of this matrix by retaining only those comparisons for which the pose tracking results showed high quality defined as a visibility score above 0.8 (based on Mediapipe outputs). We performed this filtering for each handedness separately to reduce task complexity in subsequent analyses. Additionally, we excluded gestures annotated with size or speed labels other than ‘normal,’ as these deviations could drastically influence detection results. Adaptor gestures, which are not associated with any lexemes, were also removed. These pre-processing steps resulted in three separate matrices of sizes 171  × 171 (right-handed), 128  × 128 (left-handed), and 274  × 274 (two-handed), collectively accounting for 24.15% of the original dataset. The reduction in data size was primarily driven by the stringent quality threshold for pose tracking results, which alone accounted for a 56.15% reduction.

**Fig. 7:**
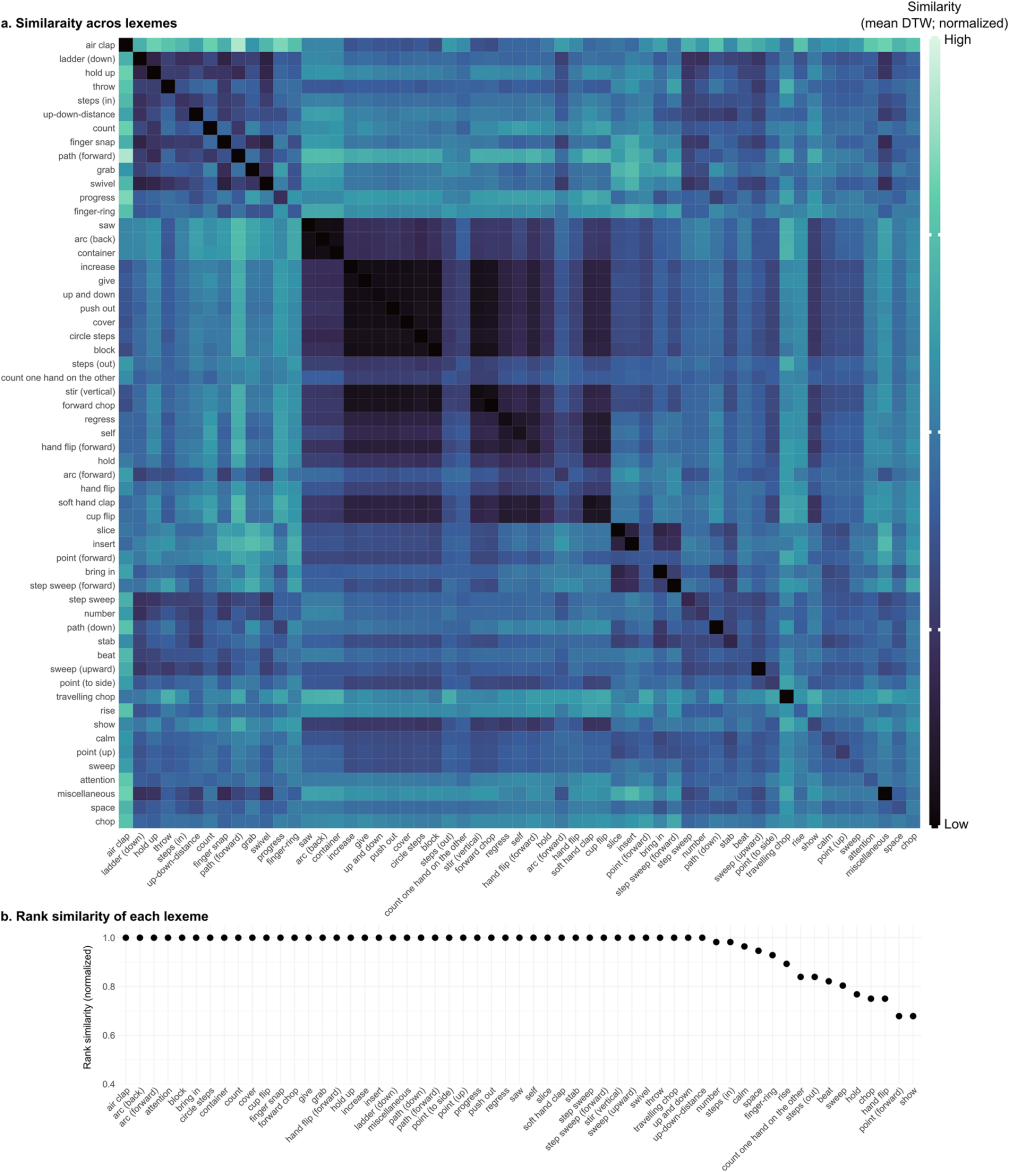
Quality of lexeme groups based on similarity of annotations’ physical features - RIGHT. (**a**). Pair-wise (dis-)similarity of lexemes (mean DTW distances across annotations). Darker colors indicate higher similarity. (**b**). Rank-similarity based on DTW values (mean across annotations), ordered from left (highest similarity) to right (lowest similarity). Higher scores correspond to higher within-group similarity.

**Fig. 8:**
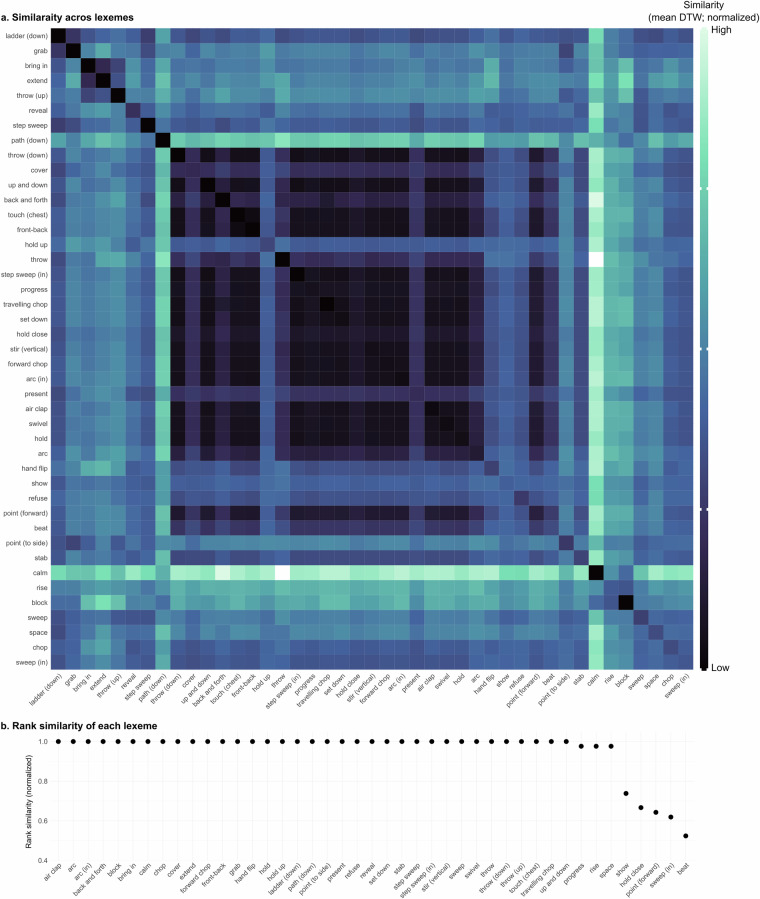
Quality of lexeme groups based on similarity of annotations’ physical features - LEFT. (**a**). Pair-wise (dis-)similarity of lexemes (mean DTW distances across annotations). Darker colors indicate higher similarity. (**b**). Rank-similarity based on DTW values (mean across annotations), ordered from left (highest similarity) to right (lowest similarity). Higher scores correspond to higher within-group similarity.

**Fig. 9:**
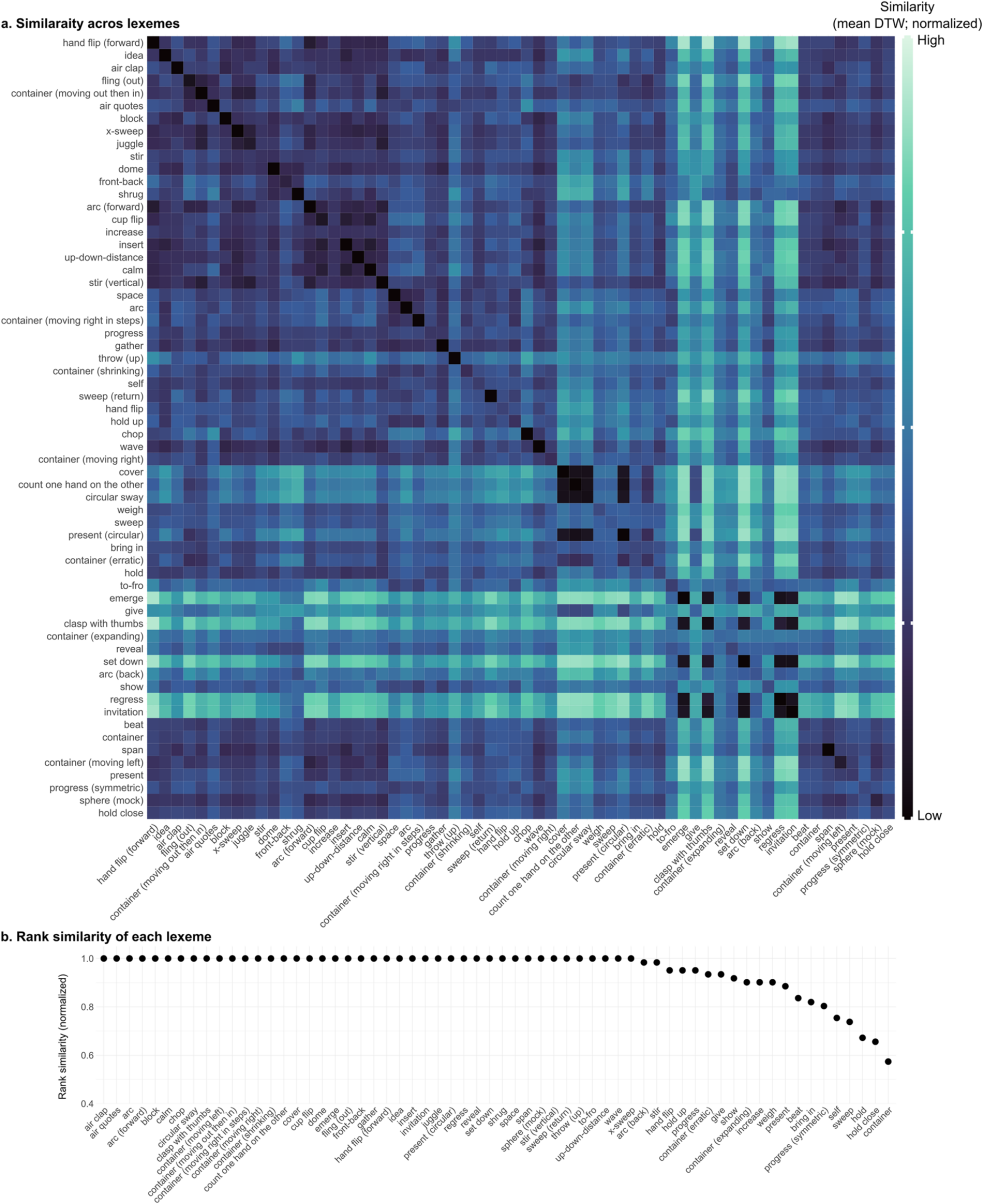
Quality of lexeme groups based on similarity of annotations’ physical features - BOTH. (**a**). Pair-wise (dis-)similarity of lexemes (mean DTW distances across annotations). Darker colors indicate higher similarity. (**b**). Rank-similarity based on DTW values (mean across annotations), ordered from left (highest similarity) to right (lowest similarity). Higher scores correspond to higher within-group similarity.

Figures 7, 8, 9a show these similarities (mean across annotations), for each handedness value separately and sorted by hierarchical clustering. Darker colors indicate higher similarity. A darker diagonal line would indicate good within-lexeme similarity. Across the three handedness values, we generally find such a dark line, with some exceptions. We then used these DTW similarity values to further explore and evaluate the lexeme groups in two different ways.

First, to evaluate and visualize the within-group similarity of the lexemes, we ordered the mean pair-wise similarity ratings to create within-lexeme rank similarities. Specifically, we averaged the DTW scores for each lexeme-pair comparison (mean across annotations). Then, for each lexeme separately, we ranked these similarity scores and extracted the rank of lexeme to same lexeme. For example, a lexeme that is most similar to itself, receives rank 1, while a lexeme that is more similar to 5 other lexemes compared to itself receives rank 6. Figures 7, 8, 9b show the rank similarity (normalized across lexemes; y-axis) for lexemes (x-axis) that occurred at least twice across the dataset and for each handedness separately. Higher values denote lexemes with higher within-lexeme similarity of 3D pose tracking data compared to other lexemes and therefore indicate physically well-defined groups of gestures. This revealed that 75.86%, 81.4%, and 64.52% of lexemes showed highest within-group similarity for right-handed, left-handed, and two-handed gestures, respectively. These results therefore indicate high although not perfect within-group similarity.

Finally, to assess whether lexeme groups are recoverable through bottom-up clustering, we performed hierarchical clustering on the raw pairwise similarity (DTW) scores of the lexemes. As before, we conducted this analysis separately for right-handed, left-handed, and two-handed gestures to reduce task complexity. We then evaluated quality of the clustering results using Normalized Mutual Information (NMI), which measures the agreement between the lexeme labels and the cluster assignments. NMI, a probability-theoretic measure, captures the mutual dependence between two variables, yielding a score between 0 and 1. Higher values indicate greater similarity between the two distributions. The clustering results showed promising NMI scores: 0.66 for right-handed, 0.55 for left-handed, and 0.50 for two-handed gestures. While these values may appear moderate, it is important to recognize that gesture classification from 3D tracking data, particularly across a large and varied lexicon of lexemes, is a highly complex task. As such, these NMI values indicate meaningful clustering performance given the difficulty of the problem. It is also worth noting that the analyses presented here are based solely on wrist positions. This means that distinctions apparent to human annotators, such as differences in handshapes, are not fully reflected in the data recovered by the tracking tool. Thus, our results underscore the value of human annotation in capturing gestural nuances not yet accessible through automated tracking methods.

## Usage Notes

We view this dataset as highly versatile and therefore envision it to be used in a variety of research fields. We expect that many potential applications of this data will become apparent in the years to come but we list here several applications that we hope to use this data for and see others use this data for:

### Gesture generation

Generating human-like gestures is challenging due to the complexity of gestures. Traditional approaches often rely on extensive manual annotations which can thus be supplemented by GESRes. However, more recent work has highlighted the potential of Large Language Models (LLMs) to generate gestures for different contexts^63,64^. Here in particular, GESRes may be valuable due to its mix between highly standardized physical descriptions of gestures and more semantically interpretable descriptions of each gesture in the form of lexemes. Recent advances show that these descriptions can be used to systematically prompt or fine-tune LLMs. Subsequently, such LLMs can predict gesture lexemes and physical properties for novel utterances. With its comparatively large set of highly varied lexemes, including cross-speaker variance, and the accompanying detailed annotations, GESRes is placed excellently to address such aims.

### Annotation automation

Another promising application is the automation of gesture annotation. As noted above, many applications such as gesture generation, require extensive annotations. Automation can significantly accelerate this process. Much work has therefore gone into developing automatic annotation processes. These can be either human-assisted^65,66^, for example requiring a small number of initial annotations for each target video, or entirely automatic once trained on an annotated dataset^67,68^. GESRes can help develop this work by adding a new set of gestures from a variety of speakers to train such annotation algorithms.

### Psychology and linguistics

Finally, this dataset may help address a wide variety of research questions in the psychological and linguistic literature. For instance, the dataset contains an unprecedented amount of metaphoric gestures. Such gestures are difficult to elicit in lab settings and therefore less well studied than, for example, iconic gestures. Here, we include 891 metaphoric gestures, many of which form gestural sequences of metaphoric gestures. Possible research questions include how the use of metaphoric gestures differs between speaker domains, across speaker culture, the physical form of metaphoric gestures, and how metaphor in language and gestures interact (e.g., co-occur or not). More generally, GESRes may help explore the link between form and function in gestures, individual similarities and differences in gesturing, and how gestures produced in different contexts are perceived by others.

### General usage notes

We have several recommendations to support effective and insightful use of this dataset. To make the most of the dataset, we encourage users to consult the ‘Lexemes.csv’ file and the codebook^54^. These resources provide detailed information on interpreting lexemes and physical properties, including asymmetric two-handed gestures. For instance, in the *space* gesture, one hand typically points to the other, which remains palm up. In such cases, handedness reflects the pointing direction (e.g., toward the right). Similarly, radial orientation can be coded as side, meaning each hand is on its respective side, or as inward to right, indicating both hands are positioned on the right side of the body.

**Gesture categories and features:** Palm orientations in this dataset are richly described to capture gesture nuance, using terms such as *in-body* or *down-front*. For instance, *down-front* refers to a palm facing forward and slightly downward. This level of detail provides opportunities for fine-grained analyses. However, depending on the goals of the analysis, simplifying some of these intermediate categories may be beneficial. For example, *down-front* could be recoded as *down* to capture broader-level similarities. A complete list of palm orientations is available in the codebook^54^. Furthermore, adaptors are included in the dataset but are not associated with lexemes. While they may be of interest in certain research contexts, we recommend excluding them from analyses focused on gesture form or meaning. Likewise, although size and speed are annotated, they show relatively little variation across the dataset, so analyses relying on these dimensions alone may yield limited insights.

**Adding new annotations:** Four of the annotated videos feature interactions with another person. In these cases, only the primary speaker was annotated. Where licensing permitted, we have included the full videos so researchers interested in social dynamics or interactional gestures can add additional annotations. We strongly encourage researchers to first familiarize themselves with the annotation manual and accompanying examples, as consistent and reliable coding depends on a solid understanding of the annotation scheme and its decision rules.

**Working with MediaPipe:** Finally, several steps of the analysis pipeline are supported by Google Colaboratory (Colab) scripts, which we provide as part of the resources on the OSF. For example, to extract hand positions and body poses using MediaPipe, we suggest using and adapting our Colab-based tools. Colab offers a convenient and accessible platform with pre-installed dependencies, enabling quick setup and streamlined execution of our code. Additionally, while MediaPipe is a robust pose detection tool, it performs best when joints and hands are fully visible. For optimal results, we recommend applying quality thresholds appropriate to your application when working with the pose tracking data.

## Data Availability

The GESRes dataset is available from the Open Science Framework (OSF)^54^ and can be accessed at 10.17605/OSF.IO/9ZKFM.

## Code availability

All code used to create this dataset and accompanying figures is available with the dataset on the Open Science Framework^54^.
